# Arbuscular Mycorrhizal Fungi Promote the Growth of *Ceratocarpus arenarius* (Chenopodiaceae) with No Enhancement of Phosphorus Nutrition

**DOI:** 10.1371/journal.pone.0041151

**Published:** 2012-09-05

**Authors:** Tao Zhang, Ning Shi, Dengsha Bai, Yinglong Chen, Gu Feng

**Affiliations:** 1 College of Resources and Environmental Sciences, China Agricultural University, Beijing, China; 2 Centre for Resource, Environment and Food Security, China Agricultural University, Beijing, China; 3 Institute of Nuclear Technology and Biotechnology, Xinjiang Academy of Agricultural Science, Urumqi, Xinjiang, China; 4 School of Earth and Environment, The University of Western Australia, Crawley, Perth, Australia; Freie Universität Berlin, Germany

## Abstract

The mycorrhizal status of plants in the Chenopodiaceae is not well studied with a few controversial reports. This study examined arbuscular mycorrhizal (AM) colonization and growth response of *Ceratocarpus arenarius* in the field and a greenhouse inoculation trial. The colonization rate of AM fungi in *C. arenarius* in in-growth field cores was low (around 15%). Vesicles and intraradical hyphae were present during all growth stages, but no arbuscules were observed. Sequencing analysis of the large ribosomal rDNA subunit detected four culturable *Glomus* species, *G. intraradices*, *G. mosseae*, *G. etunicatum* and *G. microaggregatum* together with eight unculturable species belong to the Glomeromycota in the root system of *C. arenarius* collected from the field. These results establish the mycotrophic status of *C. arenarius*. Both in the field and in the greenhouse inoculation trial, the growth of *C. arenarius* was stimulated by the indigenous AM fungal community and the inoculated AM fungal isolates, respectively, but the P uptake and concentration of the mycorrhizal plants did not increase significantly over the controls in both experiments. Furthermore, the AM fungi significantly increased seed production. Our results suggest that an alternative reciprocal benefit to carbon-phosphorus trade-off between AM fungi and the chenopod plant might exist in the extremely arid environment.

## Introduction

Plant species belonging to the Chenopodiaceae show high drought and salinity resistance and tolerance to nutrient deficiency, and often grow in psammophytic or halophytic plant communities. They are pioneer plants in colonization and settlement of harsh edaphic environments which are affected by salt or drought, and therefore play crucial roles in erosion control and rehabilitation of desert ecosystem.

Arbuscular mycorrhizas (AM) are characterized by the formation of unique structures such as arbuscules and vesicles by fungi of the phylum Glomeromycota, which have traits that are distinct from the other fungal groups [1], [2]. AM fungi are significant drivers of nutrient cycling [3], and they promote seedling establishment in degraded ecosystems [4], [5]. In some extreme environments, some plants species are unable to survive without AM fungi [6], 7. Chenopods are generally regarded as non- AM plants for the arbuscules are very rarely observed in their roots [8]. However, increasing evidence, including microscopic characterization, demonstrates that many chenopods can be well colonized by AM fungi both in the field and in pot cultures [9], [10], [11], . For example, Sengupta and Chaudhuri (1990) found high levels of colonization in two Chenopods, *Arthrocnemum indicum* and *Suaeda maritima*, in salt marshes of the Ganges delta [9]. Recently, Aleman and Tiver (2010) observed the characteristically structures of the symbionts with both arbuscules and vesicles present in some South Australian species of Chenopodiaceae [14], though their frequency of occurrence was relatively low.

Previous studies on mycorrhizal associations in the roots of chenopods have often been descriptive concerned with the colonization dynamics or characteristic structures of the symbionts without molecular identification. To our knowledge, very few studies have examined the ecological interactions between the AM fungi and chenopods. Williams *et al.* [15] first reported that inoculation with *Glomus mosseae* increased the growth of *Atriplex canescens* grown in sterilized soil. However, the growth response of chenopods to AM fungi under field conditions remains undocumented.

*Ceratocarpus arenarius* is a dominant desert annual in the Chenopodiaceae [16], [17]. It plays an important role in sand dune stabilization in the Gurbantunggut Desert, the second largest desert in central Asia [18]. Our previous observations found vesicles and hyphae but no arbuscule structures in roots of this plant species. Accordingly, we classified it as a possible AM fungi-colonized plant [12], [19]. The objectives of the present study were to verify, using molecular and microscopic methods, whether *C. arenarius* is able to be colonized by AM fungi, and to examine the growth response of *C. arenarius* to AM fungal inoculation under greenhouse and field conditions.

## Materials and Methods

### Ethics statement

All field work was approved by the Dzungaria Basin Nature Reserve Committee and performed to conform with the Regulations of the People's Republic of China on Nature Reserves. No endangered or protected species were involved in this field investigation.

### Study area

The Gurbantunggut Desert is a fixed and semi-fixed desert. It is located at the hinterland of the Dzungaria Basin (34°09′–49°08′N, 73°25′–96°24′E) in Xinjiang, northwestern China. Its annual accumulative temperature varies from 3,000 to 3,500°C. Annual precipitation is 70–150 mm with a mean of 143 mm, mainly falling in spring and autumn. During winter, the desert is usually covered by snow to a depth of more than 20 cm. Annual evaporation in this region is more than 2,000 mm. The average soil moisture content in the topsoil (top 30 cm) is 2 to 5% in April and only 0.8 to 1.2% in May in inter-dune depressions and base of the dunes [20].

A series of experiments were conducted to verify whether or not *C. arenarius* can be colonized by AM fungi and the effects of AM fungi on plant growth. Firstly, we assessed the level of colonization by AM fungi in *C. arenarius* in the field by using microscopic observation and a molecular probe method (Experiment 1). Secondly, we determined the effects of indigenous AM fungi on the growth of *C. arenarius* on-site in the Gurbantunggut Desert (Experiment 2). Thirdly, the effects of inoculation with AM fungi on the growth of *C. arenarius* were investigated under greenhouse conditions (Experiment 3).

### Experiment 1: Mycorrhizal colonization and AM fungi species in the roots of field grown C. arenarius

Root samples were collected from the Gurbantunggut Desert weekly over five weeks in spring (beginning 4 April 2009). Ten seedlings were collected at each time. Entire plants were dug out using a hand trowel, and the roots and shoots were separated in the laboratory. Shoots were oven dried at 70°C and for 72 h and the dry weights were recorded.

Fresh roots of the plants collected from the field were cut into 1-cm-long segments and thoroughly mixed. A sub-sample (ca. 0.2 g) was cleared with 10% (w/v) KOH at 90°C in a water bath for 20–30 min and stained with 0.5% (w/v) Trypan blue according to the method of Brundrett [21]. Frequency of mycorrhizas (F%), extent of root cortex colonization (M%), and abundance of arbuscules (A%) and vesicles (V%) in the root system were calculated according to Trouvelot et al. [22] using the MYCOCALC (http://www.dijon.inra.fr/mychintec/Mycocalc-prg/download.html) program. The remaining root system was cut into small pieces, oven dried overnight at 70°C, and stored in air-tight containers for subsequent AM fungal DNA processing as described by Dumbrell et al. (2011) [23].

The roots samples were ground to a homogenous powder using a ball bearing grinder. The DNA was extracted from 50 mg of mixed powder following the direction for use of the Plant Genomic DNA Kit (Tiangen Biotech Beijing Co., Ltd.). Fungal isolates were identified by the large subunit (LSU) region of ribosomal RNA-targeted nested PCR [24]. The first step was performed with the eukaryotic primer pair LR1 and NDLL22 [24], and the PCR products were further amplified in the second step with the AM fungi general primer pair FLR3 and FLR4 [25] (Table 1).

**Table 1:** PCR primers used in this study.

Primer	Sequence(5′-3′)	Target organism	Reference
LR1	GCATATCAATAAGCGGAGGA	Eucaryotic	van Tuinen et al. (1998)
NDL22	TGGTCCGTGTTTCAAGACG	Eucaryotic	van Tuinen et al. (1998)
FLR4	TACGTCAACATCCTTAACGAA	Glomeromycota	Gollotte et al. (2004)
FLR3	TTGAAAGGGAAACGATTGAAGT	Glomeromycota	Gollotte et al. 2004)

The DNA extract (5 µL) was added into the first step PCR reaction, then the product (1 µL) was used for the second-step PCR reaction. PCRs were performed in a final volume of 20 µL and the cycling conditions were as follows: initial denaturation at 93°C for 3 min, then 30 cycles with denaturation at 93°C for 40 s, annealing at 54°C for 40 s, followed by elongation at 72°C for 50 s. The cycle was finalized by elongation at 72°C for 10 min. The expected length of the PCR products was confirmed by electrophoresis in 1% (w/v) denaturing agarose gels in TAE buffer [26].

The TIANgel Midi Purification Kit (Tiangen Biotech) was used to purify the PCR products. Finally, we used the purified PCR products collected on 3 May to clone (vector pMD19-T, TA Cloning Kit for Sequencing, TAKARA) according to the manufacturer's recommended protocol. Following transformation of plasmids into host cells and blue/white screening, colonies with inserts were verified by PCR with vector–specific primers (5′-GAGCGGATAACAATTTCACACAGG and 5′-CAGCACTGACCCTTTTGGGACCGC) that flanked the cloning region (TAKARA). All the clones were picked for sequencing in the company of BGI. A set of 76 sequences were obtained from the 28s LSU rRNA region (each region around 370 bps in length). These sequences were submitted to the BLAST query tool [27] and database (www.ncbi.nih.gov/blast/) for analysis and identification of AM fungi phylotypes. Sequence alignments were performed manually. Finally, we selected 4 to 5 sequences for each phylotype, and then a phylogenetic analysis was carried out by distance analysis using the neighbor-joining method in MEGA 4 with the Kimura two-parameter model, and a gamma shape parameter of 0.1. Bootstrap analyses were done with 1,000 replications. Sequences selected from public databases belonging to known AM fungal species were included in the phylogenetic analysis with *Glomus drummondi* as the outgroup.

### Experiment 2: The in situ effects of an indigenous AM fungal community on the growth of C. arenarius in the desert

To evaluate the effects of indigenous AM fungi on the growth of *C. arenarius*, a modified in-growth core system [28] was used in the desert in 2009. Cores were constructed using polyvinylchloride (PVC) water pipe (3.2 cm inner diameter, 3.5 cm outer diameter in 20-cm sections). Two rectangular windows (8 cm length, 2 cm width) were cut symmetrically into the pipe, and the distance from window to top was 7 cm and to bottom was 5 cm. A 30-µm nylon mesh (allowing hyphal but not root penetration) was attached to the core window. The base of each pipe was sealed using a rubber plug to prevent entry by mycelia or roots.

Soil collected from the desert with the same properties as described above was sterilized with 10 k Gy ^60^Co γ-rays and then air-dried. Two hundred grams of the sterilized soil were placed into each pipe. Seeds of *C. arenarius* were collected from the desert in June 2008 and stored at −20°C before use. The seeds were surface-sterilized in 10% hydrogen peroxide for 10 min and rinsed at least 5 times in deionized (DI) water. Seeds were pre-incubated on moist filter paper for 48 h until the radicles appeared. Ten uniform seeds were sown per pipe and the seedlings were thinned to three respectively after emergence.

The experimental design was a random block with two treatments, namely rotated (prevent AM fungal colonization of the plants) and static (no restriction of AM fungi colonization of the plants). Ten blocks were randomly set in a five 2×2 m^2^ plot which was selected before the germination of the ephemeral plants to avoid any interference from heterogenity in resources or adjacent plant composition. Holes were drilled with a larger PVC tube to install the experimental pipes, and the gap between the sandy soil and the pipe wall was filled with the soil. The PVC pipes were inserted with the top 3 cm above the soil surface. In the rotated treatment the pipes were rotated approx. 45° around their vertical axes every two days in order to limit hyphal penetration of the core [28]. In the static treatment, the cores remained static to allow any indigenous AM fungal mycelium to colonize the plant roots in the pipe. Five seeds were supplemented and seedlings were thinned to one in each pipe. Plants were harvested 60 days after sowing. Mycorrhizal colonization in the root system was determined as described above. Seed number was recorded, and shoot biomass was determined after oven drying at 70°C for 48 h. Oven-dried shoots were ground and ashed in a muffle furnace at 300°C for 3 h and at 550°C for 5 h. The ash was dissolved in 2% (v/v) HCl. Phosphorus concentration was determined by inductively coupled plasma-atomic emission spectroscopy (ICP-AES; Perkin Elmer Optima 3300DV).

### Experiment 3: Effects of inoculation with indigenous AM fungi on the growth of C. arenarus under greenhouse conditions

The three dominant AM fungal species, *Glomus mosseae*, *Glomus etunicatum*, and *Glomus intradices* [19], were previously isolated from the desert soil and propagated in the soil with red clover and sorghum for 4 months in a glasshouse. The mycorrhizal inoculum consisted of root fragments, hyphae, spores (approx 200 spores in 5 g soil) and soil. In the mycorrhizal treatment each pot was inoculated with 150 g inoculum mix (a mix of all three AM fungi species at 1∶1∶1). The same amount of the inoculum mix treated with 10 k Gy ^60^Co γ-rays was used for the non-mycorrhizal treatment.

The experiment was a randomized block factorial design with two treatments, namely a mycorrhizal treatment (AM) and a non-mycorrhizal treatment (NM), each replicated 10 times. To minimize differences in the rhizosphere microbial communities between mycorrhizal and non-mycorrhizal treatments, 10 mL of filtrate free from mycorrhizal propagules from the inoculum mixture was added to each pot of the non-mycorrhizal treatment, and 10 ml of deionized water to each inoculated pot.

At the start of the experiment, 2 kg sterile sand soil combined with 150 g AM fungal inoculum was placed in the PVC pipe (20 cm tall and 10 cm in diameter, and with a PVC cap on the bottom). The seed germination rate of *C. arenarius* is relative low under laboratory conditions and we therefore transplanted seedlings directly from the field. Five-day-old seedlings with two cotyledons and 2-cm in height were collected from the desert. The seedlings were pre-selected according to height and cotyledon size. The selected seedlings were immersed into a solution of 1.5 g l^−1^ benomyl for 5 min to reduce any effect from indigenous AM fungi on the root surface [29]. Ten seedlings were transplanted into each pot and thinned to five per pot 7 days after transplanting. The pots were watered three times each week. No nutrients were added during the growth period. The plants were harvested 60 days after transplanting when as they approached maturity. Mycorrhizal colonization, P concentration, seed number and shoot biomass were determined as described above.

### Statistical analysis

Percentage data of root length colonized by AM fungi comprising colonization rate, abundance of arbuscules and vesicles, were normalized by arcsine transformation prior to statistical analysis. Data were subjected to one-way analysis of variance (ANOVA) using SPSS software version 16.0 (SPSS Inc., Chicago, IL, USA). Critical differences between pairs of mean values were compared by least significant difference (LSD) at the 5% level.

## Results

### Mycorrhizal colonization

**Figure 1:**
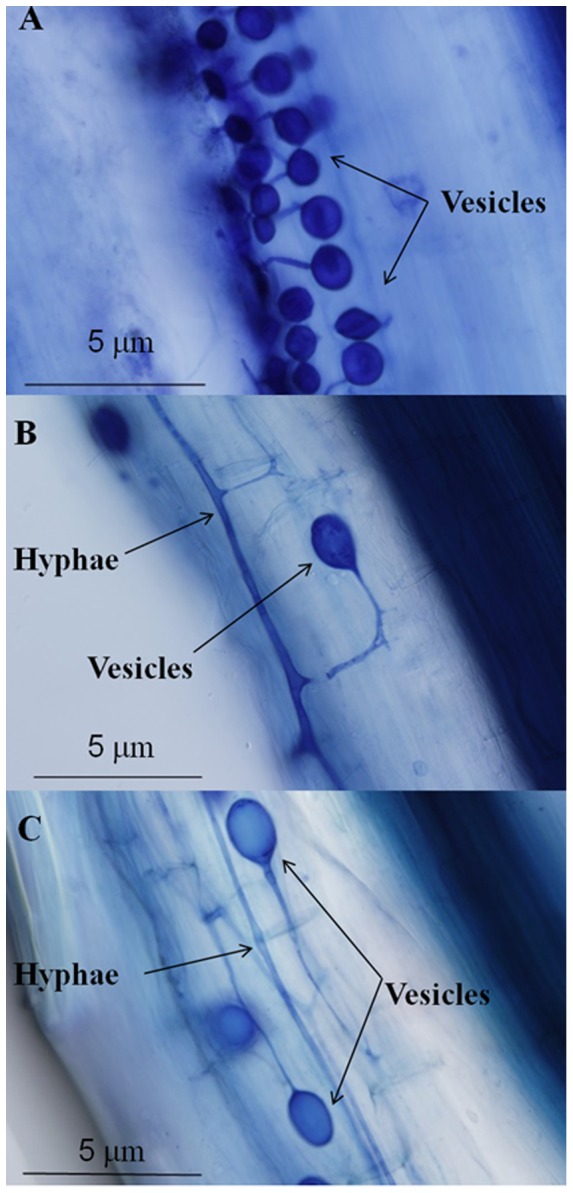
Mycorrhizal structures (×400) in roots of ***C. arenarius*** **.** A collected from Gurbantunggut Desert on 12 April 2009 in experiment 1, B from experiment 2, and C from experiment 3.

Vesicles and intraradical hyphae were observed in the root systems of *C. arenarius* (Figure 1A) collected from the field (Experiment 1) but no typical arbuscules were found. A relatively low level of colonization rate with a mean of 15% was detected from spring to summer. Significant differences were found in colonization percentage among different sampling times ranging from 10 to 20% (*P*<0.05) (Table 2). The highest colonization rate appeared at the first harvest. The extents of root cortex colonization and vesicle abundance were about 1% and 0.5%, respectively (Table 2).

**Table 2:** Dynamics of mycorrhizal colonization in the root system of *C. arenarius* from the field at different harvest times in Experiment 1.

Harvest time	F%	M%	V%
12/4	17.77±3.19a	0.40±0.11b	0.04±0.01c
19/4	11.11±3.18bc	1.02±0.41a	0.50±0.10a
26/4	12.22±2.94abc	1.07±0.21a	0.53±0.05a
3/5	15.56±1.11ab	1.15±0.18a	0.56±0.05a
9/5	10.00+1.92c	0.30±0.06b	0.25±0.02b

Different lowercase letters in each column represent significant difference (*P*<0.05) among different times.

Vesicles and intraradical hyphae were also detected in the root systems of *C. arenarius* in mycorrhizal treatments from the field experiment (Experiment 2) (Figure 1B) and the pot inoculation experiment (Experiment 3) (Figure 1C), and the presence of mycorrhiza (F%) in the roots of *C. arenarius* from mycorrhizal treatments were 3.75 and 5.25 times higher than in non-mycorrhizal treatments, respectively. There were significant differences in mycorrhizal colonization (M%) and abundance of vesicles (V%) between the mycorrhizal and non-mycorrhizal treatments (Table 3).

**Table 3:** Mycorrhizal colonization in the root system of *C. arenarius* in experiments 2 and 3.

Experiment	Treatment	F%	M%	V%
Experiment 2	Rotated	3.33±1.36b	0.07±0.07b	0.03±0.03b
	Static	12.50+2.09a	1.44±0.31a	0.68±0.19a
Experiment 3	NM	3.33±1.05b	0.12±0.04b	0.06±0.02b
	AM	17.5±2.10a	1.51±0.46a	0.73±0.23a

Different lowercase letters in each column indicate significant differences in colonization (*P*<0.05) between mycorrhizal and non-mycorrhizal treatments.

### AM fungal species in root systems of *C. arenarius* growing in the field

**Figure 2:**
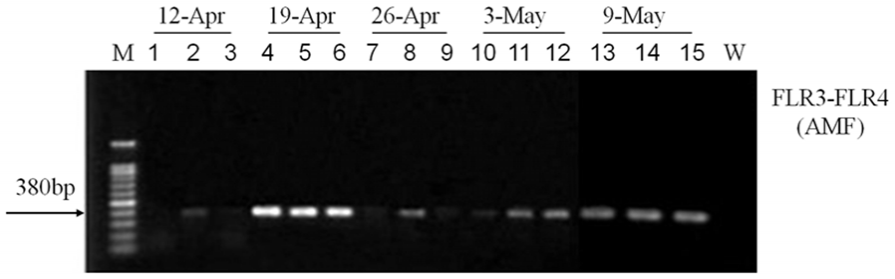
PCR detection of Glomeromycota (FLR3-FLR4) in colonized roots from experiment 1 (three replicates per sampling date). (W) indicates water control, (M) indicates molecular weight marker 100 bp ladder (Tiangen Biotech).

A PCR product of about 380 bp was amplified using the primer pair FLR3-FLR4 especially for amplifying Glomeromycota LSU rDNA sequences. In our experiment 1, the PCR detections in the period from 19 April to 9 May were successful but on others dates were less satisfactory (Figure 2). This indicates that these roots were colonized by AM fungi.

**Figure 3:**
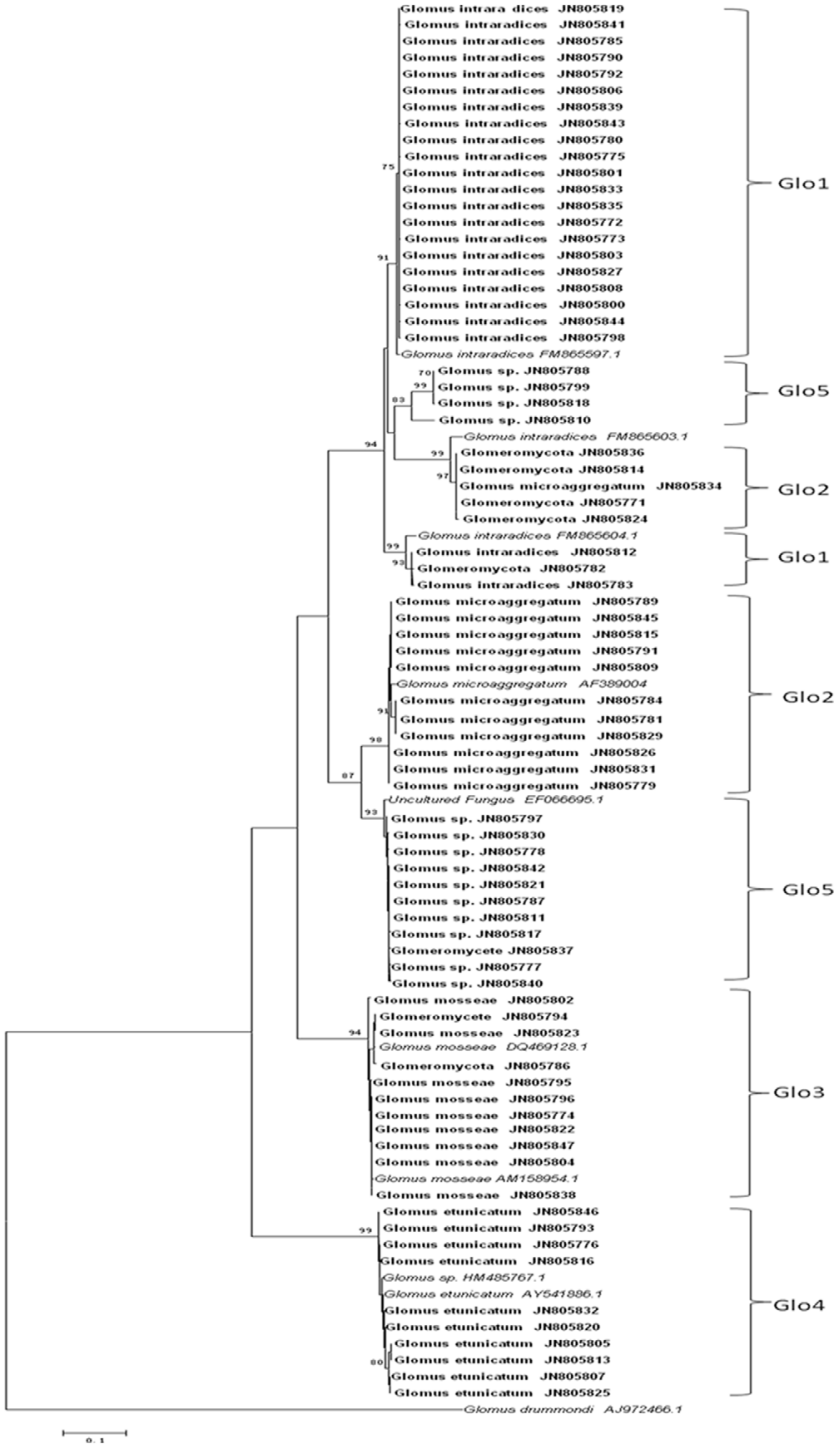
Neighbour-joining tree showing representatives of all sequence types identified in this work (in bold), and reference sequences from Genbank (in italics), using *Glomus drummondi* as the outgroup. The five topology has been tested by bootstrap analysis with 1000 replicates, and all bootstrap values >70% are shown. All new sequences have been submitted to the GenBank database (Accession nos JN805771–JN805847).

The phylogenetic analysis identified AM fungal species present in the root system of *C. arenarius* under the field condition. All the isolated AM fungi were clustered into five groups, and each sequence was matched published sequences with >97% similarity. Glo1 matched *Glomus intraradices* (GenBank Accession no. FM865597), Glo2 matched *Glomus microaggregatum* (AF389004), Glo3, *Glomus mosseae* (AM158954), Glo4, *Glomus etunicatum* (AY541886), and Glo5, while clearly *Glomus*, was not closely related to sequences from any named strains (Figure 3).

### Growth response of *C. arenarius* to AM fungi

Inoculation with the AM fungi increased the growth of the plants. In Experiment 2, the shoot biomass and seed number from static cores increased by 180% and 100% (Table 4) respectively when compared to that from rotated cores. Similarly, in Experiment 3, significant differences in shoot biomass and seed number were observed between mycorrhizal and non-mycorrhizal treatments with increments of 125% and 73.9% (Table 4) respectively, comparing with non-mycorrhizal treatments. There were no significant differences in root biomass or root/shoot ratio between mycorrhizal and non-mycorrhizal treatments in experiment 2 and 3.

**Table 4:** Shoot and root biomass, seed number, and root/shoot ratio of *C. arenarius* with or without AM fungi under field conditions and in the pot experiment.

Experiment	Treatment	Shoot biomass	Seed number	Root biomass	Root/shoot ratio
		(g plant^−1^)	(No plant^−1^)	(g plant^−1^)	
Experiment 2	Rotated	0.05±0.01b	6±1b	0.01±0.00a	0.36±0.05a
	Static	0.14±0.01a	12±2a	0.02±0.01a	0.47±0.11a
Experiment 3	NM	0.12±0.00b	23±5b	0.02±0.01a	0.13±0.01a
	AM	0.27±0.03a	40±6a	0.04±0.01a	0.16±0.04a

Different lowercase letters in each column indicate significant differences in colonization (*P*<0.05) between mycorrhizal and non-mycorrhizal treatments.

### P acquisition

**Figure 4:**
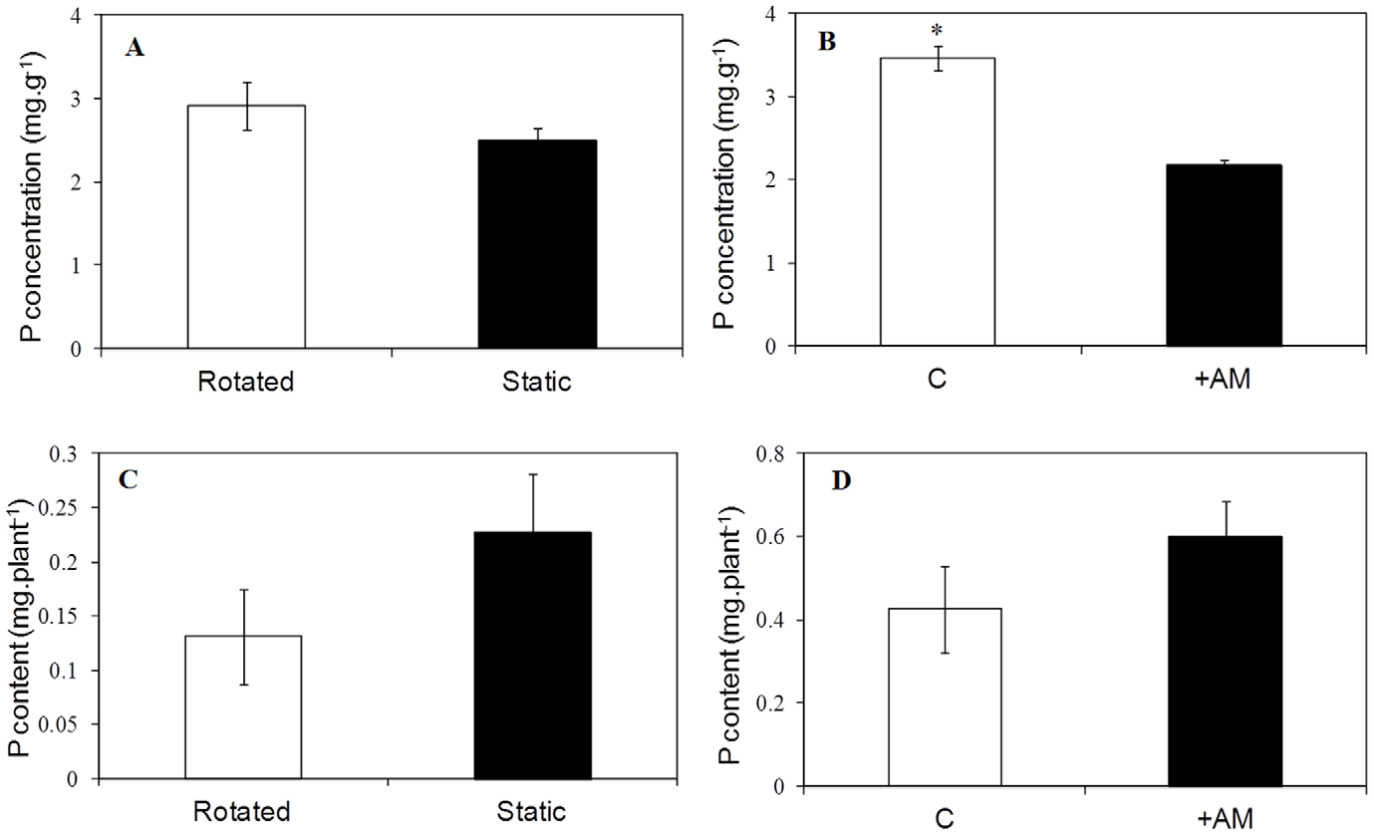
Shoot P concentration (A, B) and content (C, D) with (closed squares) or without (open squares) AM fungi in experiment 2 and 3. Asterisk indicates significant differences (*P*<0.05) between mycorrhizal treatment and non-mycorrhizal treatment.

The P content in shoot tissues of mycorrhizal plants was generally higher than that of non-mycorrhizal ones, but there was no significant difference in P acquisition in both experiments (Figure 4). In Experiment 2, there were no differences in shoot P concentration or content between rotated and static plants, respectively (both *P*>0.05; Figures 4A, 4C). In the third experiment the P concentration in plant shoots inoculated with AM fungi decreased by 37% when compared to uninoculated treatment (Figure 4B).

## Discussion

This study observed the presence of an AM fungal community in the root systems of plants *C. arenarius* plant from both the field and controlled pot experiments using microscopy and molecular probing approaches. These findings are consistent with some of early observations of AM structures (vesicles, intraradical hyphae, and even arbuscules) present in the roots of several chenopods species. Based on morphological observations, the Chenopodiaceae are considered to be a controversial plant family in terms of their mycorrhizal status. However, microscopic features of AM are often difficult to identify in AM associations in field-collected roots [8], [21], [30], and the field collected roots are often misdiagnosed [31], [32]. Our present study using LSU rDNA molecular probing confirms the presence of an AM fungal community in the roots of *C. arenarius* (Figure 3), indicating the capability of this species to form AM associations under the field and the glasshouse conditions.

Previous studies on the relationship between Chenopods and AM fungi have been mostly based on morphological description and few have explored the interactions between the two partners such as the role of the indigenous AM fungal community in the growth and nutrient uptake of Chenopods. Our present results, from both the on-site field trial and sterilized-soil pot culture, demonstrated that the indigenous AM fungal community had positive effects on the growth of *C. arenarius* (Table 4). Our results are compatible with early reports that inoculation with AM fungus increased the growth of *Atriplex canescens* [15] or *Atriplex nummularia* [33], [34] growing in sterilized soil. Asghari et al. (2005) suggested that plant age is an important factor influencing colonization percentage [33]. They found that inoculation with AM fungi decreased P concentrations at harvest after 6 weeks harvest but significantly increased P concentrations at 10 weeks, while inoculation with AM fungi significantly increased plant dry weight at 6 weeks, but not at 10 weeks.

AM symbionts generally increase plant P acquisition, and plant growth resulting from AM colonization (i.e. the ‘mycorrhizal growth response’, MGR) varies depending on the plant-fungus-environment combinations, and this is usually explained by a simple cost–benefit approach [35]. MGR may not necessarily be related to changes in the phosphorus content of plants because the mycorrhizal phosphorus uptake pathway can dominate the phosphate supply to plants irrespective of growth responses [36]. It is known that trade-off in exchange of photosynthetic carbon for soil-derived nutrients, particularly phosphorus, is a fundamental principle of symbiotic mycorrhizal associations [35], [37]. P flow at the plant-fungal interface is of benefit to the plant and also a signal to sustain the existence of the fungi in the roots. If a fungus is unable to supply P to the plant, its growth will be terminated [38]. A recent study showed that plants can detect, discriminate, and reward the best fungal partners with more carbohydrates. In turn, the fungal partners enforce cooperation by increasing nutrient transfer only to those roots providing more carbohydrates [39]. Therefore, it is clear in the present study the fungal partners delivered phosphorus to *C. arenarius* because they colonized and promoted the growth of host plant. Colonization by AM fungi did not increase the P accumulation in plant tissues (Figure 4). One possible explanation for the decrease in P concentration in the present study might be in the involvement of other mechanisms such as enhanced water uptake by the AM fungi [40]. Increased water uptake may have made a greater contribution than increased phosphorus acquisition on the growth of *C. arenarius*, with a subsequent phosphorus dilution effects in the shoots (Figure 4). Further investigation is necessary to explore the mechanisms by which the reciprocal process between AM fungi and the chenopod in addition to any effects on plant P nutrion.

Plant nutrient use efficiency is defined as biomass production per unit nutrient element (such as phosphorus) and is one of the most important functional traits for plant survival in Nature. Mycorrhizal plants with lower phosphorus use efficiency than non-mycorrhizal plants have been reported in agricultural crops [41], [42]. However, our present results indicate that phosphorus use efficiency in mycorrhizal plant is higher than in non-mycorrhizal controls in terms of lower phosphorus concentration and higher dry weight of shoots of plants colonized by AM fungi. In this context it is important to note that such a functional trait may be an important strategy for this desert plant to use a limited phosphorus resource economically in the nutrient-poor habitat.

Arbuscules have been considered as the key site of interchange of carbon and phosphorus between root cells and the hyphae of fungi of the phylum Glomeromycota, which are defining characteristics to distinguish AM fungi from other types of fungi [1]. Our molecular results verify the presence of AM fungi in the root systems of *C. arenarius*, but no arbuscules or hyphal coils were observed microscopically. These results may suggest that intraradical hyphae play a role in C/P exchange in chenopods. However, various explanations have been proposed regarding this issue. Aleman and Tiver (2010) suggested that AM fungi may not contribute greatly to the nutrient exchange between the fungi and plant roots of chenopod species in which very low presence of arbuscules was observed [14]. Asghari et al. (2005) reported that inoculation with *Glomus mosseae* lowered P concentration of *Atriplex nummularia* at the sixth week but increased the P concentration at the tenth week of growth [33]. Plenchettea and Duponnois (2005) reported that inoculation with *Glomus intraradices* increased the biomass and P concentration of *Atriplex nummularia* but no arbuscules were observed [34]. Manjarrez et al. (2010) recently showed that arbuscules are not an absolute requirement for phosphorus transfer to plants [43]. All these evidences above are implying that, beside arbuscules, an intraradical structure which is responsible for carbon/nutrients exchange must exist in Chenopods and AM fungal symbionts. More work is required to determine what factors actually control the beneficial effects of AM fungi on Chenopods.

Many studies have documented that in extremely adverse environments, such as those affected by salinity or drought habitats, Chenopods are often the dominant species in plant community. These plants follow the ‘*r*’ strategy for they have a high fecundity (particularly higher seed productivity), short generation time and an ability rapidly to exploit pulses of nutrient availability [44], [45]. Our current results show that AM fungi greatly increased the seed production of *C. arenarus*, indicating that the desert Chenopods take advantage of the local AM fungal community to explore and efficiently utilize soil resources to strengthen their ‘*r*’ strategy. Such functional traits are crucial for the species to survive in the desert habitat.

In summary, our result show that *C. arenarius*, a member of the Chenopodiaceae, was colonized by AM fungi with mycorrhizal structures presented in the root systems. Although AM fungi did not enhance the P concentration (the decline in Experiment 2 was significant as shown in Figure 4B) or uptake by the shoots, shoot biomass and plant seed production did increase significantly under both greenhouse and field conditions. Our results suggest that an alternative reciprocal process between AM fungi and the chenopod may exist in addition to C-P trade-off in the extremely arid desert environment.
